# Combining Sensory Analysis and Flavoromics to Determine How the Maillard Reaction Affects the Flavors of Golden Pomfret Hydrolysates

**DOI:** 10.3390/foods14040560

**Published:** 2025-02-08

**Authors:** Zhengsen Long, Xiangzhou Yi, Xia Gao, Yanchen Wang, Jingfeng Guo, Shuxin Gao, Guanghua Xia, Xuanri Shen

**Affiliations:** 1 Hainan Engineering Research Center of Aquatic Resources Efficient Utilization in South China Sea, Key Laboratory of Seafood Processing of Haikou, School of Food Science and Engineering, Hainan University, Haikou 570228, China; longzhengsen@outlook.com (Z.L.); xiangzhouyi1995@hainanu.edu.cn (X.Y.); gaoxia20202021@163.com (X.G.); wangyanchen@hainanuedu.cn (Y.W.); 22220951350074@hainanu.edu.cn (J.G.); 22210832000029@hainanu.edu.cn (S.G.); xiaguanghua2011@126.com (G.X.); 2 School of Food Science and Engineering, Hainan Tropic Ocean University, Sanya 572022, China

**Keywords:** Maillard reaction, golden pomfret hydrolysates, flavor, fish soups, antioxidant activity

## Abstract

Enzymatic hydrolysis can enhance the flavor of aquatic products. Nevertheless, the strong fishy odor restricts its utilization in culinary applications. This study is centered on enhancing the flavor of golden pomfret samples by promoting the Maillard reaction (MR) between golden pomfret hydrolysate (GHES) and reducing sugars. The research results demonstrate that the Maillard reaction significantly improves the sensory characteristics of GHES. It prompts the formation of diverse volatile compounds, such as aldehydes, esters, and furans. Simultaneously, it reduces the relative amounts of substances associated with fishy odor, such as 1-Octen-3-ol and Hexanal. Moreover, the Maillard reaction increases the contents of amino acids contributing to umami and sweetness, as well as 5′-nucleotides in the samples, thus enriching their umami flavor profiles. After undergoing the Maillard reaction treatment, the antioxidant capacity of the samples is also significantly enhanced (*p* < 0.05). This research highlights the potential of the Maillard reaction in improving both the flavor and antioxidant properties of GHES, establishing a theoretical basis for elevating the quality of golden pomfret products.

## 1. Introduction

The golden pomfret is acknowledged as a marine fish of significant economic value, mainly due to the absence of spines present between its muscles and its rich nutritional profile. This fish is extensively consumed and farmed along China’s southern coastline [1,2]. In 2021, the overall yield of golden pomfret reached around 243,000 tons, with its industrial worth surpassing RMB 20 billion [3]. Currently, golden pomfret is mostly offered in either fresh or frozen varieties, resulting in a limited selection of products that fails to satisfy consumer preferences for food variety, convenience, and nutritional content [4,5]. Consequently, examining the possibilities of golden pomfret soup could boost the economic significance of this species and further investigate its potential uses.

Fish soup, a staple culinary dish, is abundant in nutrients and has a unique flavor profile. Nonetheless, traditional cooking techniques, such as stewing, fail to effectively extract flavor compounds—including peptides, free amino acids, and nucleotides that contribute to taste—from the ingredients. These methods frequently lead to inadequate umami levels and overpowering fish odor, which can reduce the soup’s appeal and overall enjoyment [6,7]. Enzymatic hydrolysis offers numerous benefits, including simple reactions, mild processing conditions, and enhanced efficiency [8]. This method significantly boosts the extraction of flavor compounds by breaking down fish proteins into peptides and free amino acids, thus enhancing the umami and sweetness of the soup. Furthermore, the peptides produced from enzymatic hydrolysis demonstrate advantageous characteristics, such as anticancer, antihypertensive, and antioxidant properties [9]. However, the fish hydrolysate generated from enzymatic digestion typically retains a strong fish odor, which considerably restricts its usability as a food ingredient [10]. Consequently, it is of crucial importance to adopt appropriate strategies to optimize the flavor and functional characteristics of golden pomfret hydrolysates (GHESs) and further fully explore their potential applications in the research and development of golden pomfret fish soup products.

The Maillard reaction (MR) is characterized as a non-enzymatic browning phenomenon where amino compounds and carbonyl groups engage with one another, resulting in a diverse range of melanoidins and volatile substances via processes including addition, condensation, rearrangement, and polymerization [11]. This reaction effectively enhances the flavor profile of hydrolysates and is vital for the flavor, texture, and color characteristics of processed foods [12]. A variety of aromatic compounds produced during the MR involving amino acids, peptides, or proteins interacting with sugar carbonyls can reduce and conceal fish odors, thus increasing overall acceptability [13]. Additionally, the MR not only amplifies food product flavors but also enhances the functional aspects of hydrolyzed proteins and elevates antioxidant activity [14]. The electron transfer properties of hydroxyl and pyrrole groups found in MR products (such as Amadori derivatives, reduced ketones, and heme-like substances), in conjunction with the hydrogen potential from these reduced ketones, can interrupt free-radical chain reactions. Through activities like metal chelation, decomposition of H_2_O_2_, and trapping of reactive oxygen species, these compounds provide considerable antioxidant benefits [15].

The purpose of this study is to improve the flavor of GHES by promoting the MR between GHES and reducing sugars, so as to provide a theoretical basis for the application of GHES in fish soup. We utilized ultraviolet-visible spectroscopy, fluorescence spectroscopy, and Fourier transform infrared spectroscopy to analyze protein degradation and the onset of the MR. Sensory evaluation, alongside flavoromics techniques, was employed to evaluate how the processing influenced the flavor characteristics of the golden pomfret products. Moreover, we investigated the relationships between aroma descriptors and key volatile compounds using partial least squares regression (PLSR). In addition, we assessed the antioxidant activity after MR treatment to determine the biological effects of the GHES.

## 2. Materials and Methods

The local market in Haikou, China, provided the golden pomfret. Flavored protease and xylose were obtained from Henan Wanbang Chemical Technology Co., located in Zhengzhou, (Henan), China. All other reagents utilized in this research were of analytical quality and sourced from the Guangzhou Chemical Reagent Factory in Guangzhou, China.

### 2.1. Determination of Basic Chemical Components of Golden Pomfret Fish

The golden pomfret fish was homogenized using a blender following the removal of viscera and fins, and the minced meat was analyzed for moisture, protein, fat, and total sugar according to the method referenced in [16]. All measurements were carried out in triplicate.

### 2.2. Sample Preparation

To ensure the consistency and comparability of experimental conditions, the golden pomfret for the three treatment groups, namely Stewed golden pomfret samples (Control), golden pomfret hydrolysates (GHESs), and the products by facilitating the Maillard reaction (MR) between GHES and xylose (MRPS), were all sourced from the same batch.

Control: Golden pomfret, obtained from a nearby market located in Haikou, China, was prepared by excising the viscera and fins. A total of 200 g of fish was minced and combined with water at a 1:3 mass-to-volume ratio, then subjected to heating in a water bath at a temperature of 50 °C for a duration of 8 h. Following this, a heating phase of 15 min was conducted at 100 °C. Ultimately, it was centrifuged, and the supernatant was preserved at −20 °C for future applications. Three specimens were prepared for each group of samples. Each specimen was tested once, and the average value was taken for further analysis.

GHES: The conditions for preparing GHES were determined based on earlier preliminary tests (see Supplementary Table S1). The degree of hydrolysis serves as an indicator of peptide bond disruption and a reduction in the molecular weight of peptides, making it a widely utilized assay for assessing protein hydrolysis. Table S1 presents the hydrolysis of golden pomfret by five proteases under varying conditions. Notably, the degree of hydrolysis of golden pomfret exhibited significant differences across different pH levels (*p* < 0.05), with an optimal pH of 7 for all five proteases. Among them, flavored protease demonstrated the highest degree of hydrolysis at 23.30 ± 0.41%. Neutral protease achieved its maximum hydrolysis at 55 °C (17.75 ± 0.47%), while the optimal temperature for the other four enzymes was 50 °C, with flavored protease showing the highest degree of hydrolysis at 23.20 ± 0.56%. A one-way analysis of enzyme addition indicated that the degree of hydrolysis increased with the amount of enzyme added. However, when the enzyme concentration reached 0.8% (*w*/*v*), further increases did not lead to a significant change in hydrolysis (*p* < 0.05), indicating that 0.8% is the most suitable enzyme concentration. Additionally, the degree of hydrolysis of golden pomfret increased with prolonged enzyme digestion time, but no significant changes were observed after 8 h (*p* < 0.05), establishing 8 h as the optimal digestion time. In summary, the maximum degree of hydrolysis for golden pomfret was achieved under the following conditions: flavored protease at 0.8% (*w*/*v*), enzymatic temperature of 50 °C, pH of 7, and an enzymatic digestion time of 8 h. In particular, 200 g fish flesh was combined with water at a 1:3 (*w*/*v*) ratio, followed by an adjustment of pH to 7.0 using 1 mol/L food-grade NaOH and HCl. Then, 0.8% (*w*/*v*) flavored protease (Henan Wanbang Chemical Technology Co., Ltd., 20,000 μ/g) was incorporated. The resultant mixture was heated in a water bath at a temperature of 50 °C for 8 h. Once this duration was completed, the temperature was raised to 100 °C to inactivate the protease for 15 min. Ultimately, it was centrifuged, and the supernatant was preserved at −20 °C for future applications. Three specimens were prepared for each group of samples. Each specimen was tested once, and the average value was taken for further analysis.

MRPS: Preliminary experiments (see Supplementary Table S2) defined the conditions for the MR. The amount of reducing sugar, reaction time, pH, and reaction temperature are critical factors influencing the MR. Researchers employed a one-factor stepwise optimization method, using sensory scores as a criterion to identify optimal conditions for the MR. As illustrated in Table S2, the sensory scores of golden pomfret soup exhibited a trend of initially increasing and then decreasing with rising pH levels, with the highest score recorded at pH 7. Consequently, pH 7 was selected as the optimal condition for further refining the fish soup preparation process. Similar trends were observed in the one-way experiments concerning reaction time, reaction temperature, and xylose addition; sensory scores also increased and then decreased, with optimal conditions determined to be a reaction time of 2 h, a reaction temperature of 120 °C, and a xylose addition of 8% (*w*/*v*). In summary, the optimal conditions for the MR were established as pH 7, a reaction time of 2 h, a reaction temperature of 120 °C, and a xylose addition of 8% (*w*/*v*). Even after adjusting the pH of 200 mL GHES to 7 using 1 mol/L food-grade NaOH and HCl, GHES with 8% (*w*/*v*) xylose was mixed in a sealed reaction bottle and reacted in an oil bath at 120 °C for 2 h. Three specimens were prepared for each group of samples. Each specimen was tested once, and the average value was taken for further analysis.

### 2.3. Sensory Evaluation

#### 2.3.1. Sensory Sample Preparation

Preparation of samples for sensory analysis was conducted by pouring 25 mL portions into 30 mL black polypropylene cups with lids. Each sample was blinded with three-digit random numbers. The samples were stored for 2 h at 25 °C to obtain a serving temperature of 25 °C.

#### 2.3.2. Sensory Profiling

The sensory evaluation experiments for the three groups of samples, namely Control, GHES, and MRPS, were conducted following the methods detailed in Reference [7], with some modifications. A sensory assessment team consisting of 10 assessors, aged between 20 and 25 years, included an equal number of 5 males and 5 females, all of whom underwent standardized screening and received professional training. Samples were assessed at a regulated temperature of 25 ± 1 °C, concentrating on characteristics such as color, fish odor, fish flavor, umami, and sweetness. The average sensory evaluation scores were then computed. A 10-point scale was employed to classify the sensory attributes, where scores of 9–10 represented excellent quality, 6–8 indicated good quality, 4–5 reflected average quality, and 1–3 represented poor quality. Each sample was evaluated in triplicate to guarantee reliability, and the average value was taken for further analysis.

### 2.4. Ultraviolet (UV) Absorption Spectroscopy

The UV absorption spectra were determined using the methodology outlined in Reference [17]. The samples were diluted to ensure that the absorbance values remained within the instrument’s operational limits, achieving a final concentration of 1 mg/mL. These diluted samples were subsequently analyzed with a UV spectrophotometer (UV-1800 PC, Beijing Pudian General Instrument Co., Ltd., Beijing, China), measuring absorbance values across a wavelength range of 190 to 500 nm, with deionized water utilized as the blank. Three specimens were prepared for each group of samples. Each specimen was tested once, and the average value was taken for further analysis.

### 2.5. Fluorescence Spectrometry

The procedure for determining the fluorescence spectra was carried out as outlined in Reference [18]. Samples were diluted with distilled water until achieving a final concentration of 1 mg/mL. A fluorescence spectrophotometer (Thermo Fisher Shanghai Instrument Co., Ltd.; Shanghai, China) was utilized to record the fluorescence spectra, employing an excitation wavelength of 347 nm and scanning the emission spectrum from 360 nm to 550 nm. Three specimens were prepared for each group of samples. Each specimen was tested once, and the average value was taken for further analysis.

### 2.6. Fourier Transform Infrared Spectroscopy (FTIR)

A total of 0.01 g of lyophilized substance was mixed with 0.5 g of KBr, which was then shaped into flakes. The infrared absorption spectra for the samples were measured across a wavelength range from 4000 to 400 cm^−1^ employing an FTIR, using KBr flakes as reference blanks. Three specimens were prepared for each group of samples. Each specimen was tested once, and the average value was taken for further analysis.

### 2.7. Color Measurement

A transparent glass container was used to transfer 10 mL from each sample, followed by the measurement of the L*, a*, and b* values with a colorimeter (model XD-200, produced by Shanghai Modern Environmental Engineering Technology Co., Ltd., Shanghai, China). Three specimens were prepared for each group of samples. Each specimen was tested once, and the average value was taken for further analysis.

The following equation was used to calculate the chroma (c*):

(1) $$c^{*}=\sqrt{{a^{*}}^{2}+{b^{*}}^{2}}$$

### 2.8. Determination of Volatile Compounds

Volatile compounds were extracted from Solid-Phase Microextraction (SPME) fiber heads, which feature a thickness of 75 μm and consist of divinylbenzene/carb-oxene/polydimethylsiloxane. A 2.0 g specimen was placed into a 10 mL headspace vial that was equilibrated at 50 °C for 5 min. The SPME fiber head was then exposed to the headspace of the sample for 25 min. After the extraction was completed, the SPME fibers were desorbed at 250 °C for 7 min into the gas chromatography (GC) feed port using a capillary DB-WAX column (30 mm × 0.25 mm × 0.25 μm film thickness, J & W Scientific, Folsom, CA, USA). Gas chromatography analysis was conducted on a TSQ Quantum XLS (Thermo Fisher Scientific Inc., Waltham, MA, USA) under the following conditions: The temperature program for the column started at 50 °C with no initial hold, was increased to 80 °C at a rate of 8 °C/min, and was maintained for 2 min. The temperature was then raised to 140 °C at 5 °C/min and held for 3 min. Afterward, the temperature was increased to 170 °C at a rate of 5 °C/min, was maintained for 5 min and then elevated to 230 °C within 1 min, where it remained for an additional 3 min. Three specimens were prepared for each group of samples. Each specimen was tested once, and the average value was taken for further analysis.

The overall flavor profile was assessed by evaluating the contribution of each ingredient through the relative odor activity value (ROAV) method, aimed at pinpointing the unique flavor compounds. The calculation of the ROAV was performed using the formula provided below:

(2) $${ROAV}_{i}=100\times \frac{C_{i}}{\;C_{max}\;}\times \frac{T_{max}}{T_{i}}$$

C_i_: relative concentration of the examined chemical; T_i_: the relevant sensory threshold of the evaluated chemical; C_max_: the relative concentration of the chemical that predominantly influences the overall flavor; T_max_: the sensory threshold of the chemical that most significantly influences the overall flavor.

### 2.9. Determination of Free Amino Acids (FAAs)

The method for determining free amino acids was provided by the Institute of Environment and Plant Protection, Chinese Academy of Tropical Agricultural Sciences. To be more specific, sample pretreatment consists of multiple steps aimed at facilitating precise analysis. Start by measuring 0.2 mL of the sample into a centrifuge tube, ensuring to record its weight. Subsequently, incorporate 1.8 mL of a 0.006 mol/L hydrochloric acid solution, which is created by diluting 1 mL of 6 mol/L hydrochloric acid in 1 L of water. Subject this mixture to ultrasonic extraction for 30 min, followed by refrigeration of the centrifuge tube at 4 °C for 8 h. After this, centrifuge the sample at 10,000 rpm for 10 min, collect the supernatant, and filter it through a 0.22 µm membrane before transferring it to a liquid-phase vial. After the initial spin, add 75 µL of a 0.1 mol/L phenyl isothiocyanate-acetonitrile solution along with 75 µL of a 1 mol/L triethylamine-acetonitrile solution. Vortex this mixture for 1 min and then incubate it in a water bath at 25 °C for a duration of 60 min. Next, introduce 1.4 mL of n-hexane, vortex for 15 s, allow the solution to settle for 2 min, and centrifuge again at 10,000 rpm for 5 min, repeating the extraction twice. Aspirate the bottom layer of the solution, utilize a 0.22 µm filter, and transfer the solution into a chromatographic injection vial for subsequent analysis. Chromatographic separation was executed using a Waters Acquity UPLC H-Class system adorned with a PDA detector and an Agilent HC C18 150 mm column, operating at a detection wavelength of 254 nm. The binary mobile phase comprised a 0.05 mol/L sodium acetate buffer trihydrate (A) and a blend of acetonitrile, methanol, and water in a 6:2:2 (*v*/*v*) ratio (B). The linear gradient elution proceeded as follows: from 0 to 8 min, 95–87% A; from 8 to 18 min, 87–82% A; from 18 to 25 min, 82–85% A; from 25 to 33 min, 65–45% A; from 33 to 40 min, 45–95% A; concluding with 40–45 min at 95% A, yielding a total duration of 45 min. The column oven temperature was maintained at 35 °C, with an injection volume fixed at 2 μL. The identification and quantification of the compounds were determined based on the peak times and areas derived from the external standard spectra. Three specimens were prepared for each group of samples. Each specimen was tested once, and the average value was taken for further analysis.

### 2.10. Determination of 5′-Nucleotides

The treatment of samples involved taking 0.2 mL from each specimen, which was then weighed. Following this, 1.8 mL of 5% trifluoroacetic acid was introduced, and the mixture underwent ultrasonication for a duration of 15 min. Subsequently, centrifugation was performed at a speed of 10,000 rpm for 3 min, and the supernatant was carefully collected. The collected supernatant was filtered using a 0.22 µm membrane and then placed into liquid-phase vials for further analysis. A Waters Acquity UPLC H-Class system with a PDA detector was employed for chromatographic analysis. The chromatographic separation was carried out using an XDB C18 column designed for atmospheric pressure (4.6 mm × 250 mm), with a detection wavelength set at 254 nm. The binary mobile phase was composed of 0.01 M dipotassium hydrogen phosphate at pH 3.69 (A) and 100% methanol (B), operating at a flow rate of 1 L/min. The linear gradient elution program was structured as follows: from 0 to 12 min, 100% A; from 12 to 15 min, transitioning from 100% to 50% A; from 15 to 20 min, holding at 50% A; from 20 to 21 min, transitioning back from 50% to 100% A; and from 21 to 25 min, returning to 100% A. The overall analysis time was 25 min. The column oven temperature was kept constant at 30 °C, and the injection volume used was 2 μL. The identification and quantification processes relied on the retention times and peak areas correlating to the external standard spectra. Three specimens were prepared for each group of samples. Each specimen was tested once, and the average value was taken for further analysis.

### 2.11. Calculation of Equivalent Umami Concentration (EUC)

The EUC values were computed using the methodology described in [19] as follows:

(3) $$EUC(g/100g)=\sum a_{i}b_{i}+1218\left(\sum a_{i}b_{i}\right)\left(\sum a_{j}b_{j}\right)$$

*a_i_*: concentration of amino acids such as Glutamate (Glu) or Aspartate (Asp); *b_i_*: concentration of amino acid umami in relative terms (Glu, 1; Asp, 0.077); *a_j_*: concentration of nucleotides including IMP, GMP, or AMP; *b_j_*: relative umami of nucleotide concentration (IMP, 1; GMP, 2.3; AMP, 0.18); 1218 represents a constant that indicates synergy.

### 2.12. Determination of Organic Acids

During the pretreatment stage, a 0.1 mL sample was measured, followed by the addition of 0.9 mL of a 0.1% aqueous phosphoric acid solution. This blend underwent ultrasonic extraction for 15 min at a frequency of 10,000 rpm. The supernatant was then gathered, passed through a 0.22 µm filter membrane, and placed into liquid-phase vials with pre-prepared caps for subsequent online analysis. Chromatographic evaluation was carried out using a Waters Acquity UPLC H-Class system outfitted with a PDA detector. For the chromatographic separation, an Agilent XDB C18 column (150 mm, Agilent, Santa Clara, CA, USA) was utilized, with the detection wavelength calibrated to 210 nm. The mobile phase consisted of 0.01 M potassium phosphate at pH 2.32 (component A) and 100% methanol (component B), flowing at a rate of 0.6 L/min. The elution gradient was orchestrated as follows: from 0 to 16 min, maintaining 100% A; from 16 to 18 min, transitioning from 100% to 80% A; from 18 to 24 min, sustaining 80% A; and finally, from 24 to 25 min, reverting back to 100% A, culminating in a total duration of 25 min. The temperature of the column was kept constant at 25 °C, with an injection volume fixed at 2 µL. The identification and quantification of the analytes were determined based on their retention times and peak areas obtained from external standard spectra. Prepare three specimens for each group of samples. Test each specimen once, and the average value was taken for further analysis.

### 2.13. Determination of Antioxidant Activity

#### 2.13.1. DPPH Free Radical Scavenging Capacity

The methodology described in reference [20] underwent slight modifications for the current study. Vc (acting as a positive control), a control group, GEHs, and MRPs were prepared in varying concentrations of 10 mg/mL, as well as 20, 30, 40, and 50 mg/mL, utilizing distilled water as the solvent. Before conducting the experiment, the DPPH solution’s absorbance was calibrated to 0.70 ± 0.02. Test samples were generated by combining 2 mL of the prepared solution with 2 mL of a 0.2 mM DPPH solution. The DPPH solution was promptly added, mixed thoroughly, and then allowed to rest in darkness for 30 min at a temperature of 25 °C. Subsequent absorbance readings were recorded at a wavelength of 517 nm. Three specimens were prepared for each group of samples. Each specimen was tested once, and the average value was taken for further analysis.

The evaluation of DPPH radical scavenging activity was performed using the following equation:

(4) $$DPPH\;radical\;scavenging\;activity\left(\%\right)=\left(1-\frac{A_{1}-A_{2}}{A_{0}}\right)\times 100\%$$

The absorbance measurement of the blank control is referred to as A_0_; A_1_ signifies the absorbance measurement of the sample; and A_2_ represents the absorbance measurement of the combination of anhydrous ethanol with the sample.

#### 2.13.2. ABTS Free Radical Scavenging Capacity

The preparation of the ABTS solution involved mixing 7 mM ABTS with 2.45 mM potassium persulphate, which was then left to incubate in the dark at 25 °C for 12 h. To initiate the reaction, 1 mL of the sample solution was added to 4 mL of the ABTS solution, and the two mixtures were thoroughly blended. After a 6 min incubation in a dark environment at 25 °C, the absorbance was recorded at 734 nm [17]. Three specimens were prepared for each group of samples. Each specimen was tested once, and the average value was taken for further analysis.

The capacity of ABTS to scavenge free radicals was evaluated using the equation provided below:

(5) $$ABTS\;radical\;scavenging\;activity\left(\%\right)=\left(1-\frac{A_{1}-A_{2}}{A_{0}}\right)\times 100\%$$

A_0_: absorbance measurement of blank control; A_1_: absorbance measurement of sample; A_2_: absorbance measurement of anhydrous ethanol and sample combination.

#### 2.13.3. Hydroxyl Radical Scavenging Capacity

In conclusion, a mixture was prepared by combining 0.6 mL of 8 mM FeSO_4_ with 0.5 mL of 20 mM H_2_O_2_ along with 2 mL of samples at different concentrations. Subsequently, 0.5 mL of a 3 mM ethanolic salicylic acid solution was added to trigger the reaction. To this reaction mixture, 0.9 mL of distilled water was incorporated, and the resulting solution was centrifuged for 10 min at 10,000 rpm. Before this centrifugation step, the mixture was incubated in a water bath set at 37 °C for 30 min to allow precipitation. The absorbance of the resulting supernatant was later assessed at 510 nm. Three specimens were prepared for each group of samples. Each specimen was tested once, and the average value was taken for further analysis.

The scavenging capacity for hydroxyl radicals was calculated using the specified equation below:

(6) $$Radical\;scavenging\;capacity\left(\%\right)=\left(1-\frac{A_{1}-A_{2}}{A_{0}}\right)\times 100\%$$

A_0_: Measurement of absorbance for the blank control; A_1_: Measurement of absorbance for the sample; A_2_: Sample that does not contain salicylic acid.

#### 2.13.4. Ferric Ion-Reducing Capacity

The capacity of ferric ion reduction was assessed utilizing a modified approach based on the methodology outlined in [21]. In this process, a volume of 800 μL of the sample solution was combined with 2 mL of a phosphate buffer solution at a concentration of 200 mM and pH 6.6, along with 2 mL of a potassium ferricyanide solution at 1% *w*/*v*. The resulting mixture was stirred vigorously for roughly 1 min and then incubated in a water bath maintained at 50 °C for a duration of 20 min. Following the incubation, 2 mL of a 10% (*w*/*v*) TCA solution was introduced into the mixture and vortexed for about 1 min. Next, a solution of ferric chloride (1 mL) was mixed with 1 mL of distilled water and 2 mL of the incubation mixture, yielding a final ferric chloride concentration of 0.1% by weight. After a subsequent incubation period of 10 min, the absorbance was recorded at 700 nm, where the reduction potential was reflected by an increase in the absorbance of the reaction mixture. Three specimens were prepared for each group of samples. Each specimen was tested once, and the average value was taken for further analysis.

### 2.14. Data Analysis

Continuous variables are expressed as mean ± standard deviation. Data analysis utilized SPSS statistical software (R23.0.0.0), applying ANOVA along with Duncan’s multiple comparisons test to assess statistical significance at *p* < 0.05. PLS-DA and clustering heatmaps were assessed using OmicShare(a website platform, the address is https://www.omicshare.com/), with the heatmaps normalized and samples organized according to the Euclidean distance method. The PLSR analysis was performed using The Unscrambler X (10.4).

## 3. Results and Discussion

### 3.1. Basic Components of Golden Pomfret

**Figure 1:**
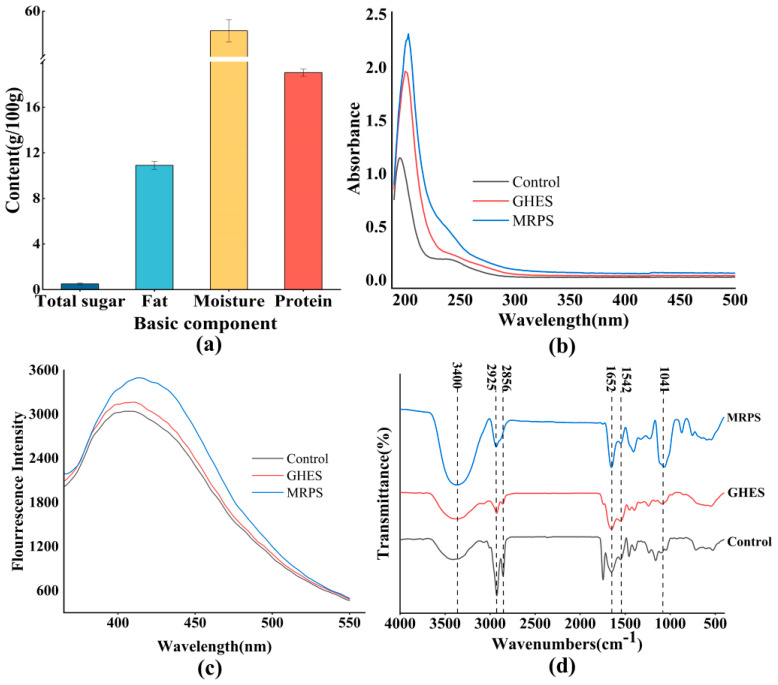
(**a**) Basic components of golden pomfret (g/100 g); (**b**) ultraviolet spectra, (**c**) fluorescence spectra, (**d**) Fourier transform infrared spectra (%) of three different golden pomfret samples. Control: stewed golden pomfret samples; GHES: golden pomfret hydrolysate; MRPS: the products by facilitating the Maillard reaction (MR) between GHES and xylose.

Proteins derived from fish have been acknowledged for their significant nutritional benefits for a long time. When compared to proteins from land animals, those from aquatic sources demonstrate enhanced digestibility and are rich in a range of peptides and essential amino acids, especially methionine and lysine, which are typically found in lower amounts in meat from terrestrial animals [22]. Previous research has shown a strong connection between the protein levels in raw materials and the formation of appealing flavors during food processing [16]. As illustrated in Figure 1a, the fundamental chemical makeup of golden pomfret comprises 58.28 ± 0.97 g/100 g of moisture, 19.03 ± 0.31 g/100 g of protein, 10.9 ± 0.36 g/100 g of fat, and a minimal total sugar content of 0.5 ± 0.08 g/100 g. These findings suggest that moisture and protein are the main chemical components of golden pomfret. The elevated protein level not only enhances the nutritional value of the associated processed foods but also supplies substantial precursors for creating enticing aromas.

### 3.2. Spectral Analysis

Figure 1b shows the UV absorption spectra for three different sample groups within the wavelength range of 190 to 500 nm. The Control, GHES, and MRPS groups demonstrated peak absorption at 195, 200, and 203 nm, respectively. Prior research has suggested that absorption in the 190–210 nm interval corresponds to peptide bonds in proteins [23]. Typically, enzymatic degradation causes these maximum absorption peaks related to peptide bonds to shift toward longer wavelengths, combined with a rise in UV absorption levels [24]. The UV absorption detected within the 260–280 nm range is mainly related to chromophores, specifically the residues of tryptophan and tyrosine. The increased UV absorption in the GHES group might be due to enzymatic modifications in the protein structure that reveal internal chromophore groups [25]. Additionally, the UV absorption spectrum reflects the production of MR intermediates and melanoidin [26]. The absorption observed between 240–300 nm is linked to Schiff base formation [27], while the peak seen at 420 nm corresponds to browning compounds created via the MR [28]. Noteworthy alterations in MRPS within the 240–300 nm region and at 420 nm signify the generation of Schiff bases and browning products.

Fluorescence spectroscopy serves as a commonly employed method for analyzing the MR. In the early phases of the MR, fluorescent substances are generated, which later convert into browning agents as the reaction continues [29]. Typically, the fluorescent outcomes of the MR can be detected in the wavelength range of 400–600 nm, with an excitation wavelength of 347 nm [30]. Figure 1c depicts the differences in fluorescence spectra across three groups of samples. The order of fluorescence intensity observed was MRPS > GHES > Control, with GHES exhibiting a minor increase in fluorescence intensity when contrasted with the Control group. This enhancement may stem from the creation of fluorescent substances during the hydrolysis process, as supported by findings from [31]. The fluorescence intensity recorded for MRPS was notably greater than that of the GHES group, suggesting that the MR was occurring along with the synthesis of fluorescent substances [32]. These fluorescence compounds might include Schiff bases associated with electron-donating groups, nitrogen-containing species, or cyclic unsaturated carbonyls [33].

Figure 1d presents the infrared spectra for the three sets of samples. The absorption peak observed near 3400 cm^−1^ is linked to the tensile stress-induced stretching vibration of O-H. This peak is significantly enhanced by MRPS at this particular wavelength when compared to GHES, likely due to the addition of sugar molecules that intensify the stretching vibrational absorption of free hydroxyl groups [34]. This effect may also be associated with the creation of extra free -OH groups following the MR. In Reference [31], a relationship is indicated between the enhancement of the absorption peak near 3400 cm^−1^ after MR and the increase in aldehydes and esters. The spectral interval of 3000–2800 cm^−1^ represents the hydrophobic regions in proteins, which are attributed to C-H stretching vibrations. The peak observed at 2925 cm^−1^ in the Control group relates to the asymmetric CH_2_ stretching vibrations found in the aliphatic side chains of proteins [35]. The noted decline in blue shift and CH_2_ intensity in GHES (2927 cm^−1^) compared to the Control (2925 cm^−1^) suggests that enzymatic hydrolysis disrupts the hydrophobic regions of the gold pomfret protein [36]. Moreover, the change in the MRPS absorption peak at 2925 cm^−1^ indicates that the interaction between gold pomfret proteins and reducing sugars results in the loss of original functional groups and the emergence of new functional groups [37]. The clear protein absorption peak at 1652 cm^−1^ corresponds to the amide I (C=O stretching vibration), while the peak at 1542 cm^−1^ represents the N-H bending vibration. In addition, the absorption peak at 1041 cm^−1^ is associated with the C-O stretching vibration [38]. The heightened intensity of the GHES absorption peaks observed at 1652 cm^−1^, 1542 cm^−1^, and 1041 cm^−1^ signifies the breakdown of proteins [20]. A notable decrease in the intensity of the N-H absorption for MRPS at 1542 cm^−1^ can be attributed to the interaction between the carbonyl group and the free amino group, leading to a modification in the structure of the peptide chain. Additionally, the marked rise in MRPS at 1041 cm^−1^ is linked to the C-O stretching prompted by xylose molecules.

Investigations using UV, fluorescence, and infrared spectroscopy demonstrated that enzymatic degradation occurred in gold pomfret, leading to alterations in its protein structure and the production of MR intermediates and browning compounds upon the addition of xylose and an increase in temperature.

### 3.3. Color and Sensory Evaluation

Color is essential in influencing how consumers perceive products, greatly affecting their choices and buying behaviors [39]. The results from colorimeter measurements for three sample groups are shown in Table 1. There was an increase in the L* value, while the a* value decreased in GHES when compared to the Control (*p* < 0.05), which aligns with the findings noted by [40]. After enzymatic hydrolysis, the b* value showed a significant rise (*p* < 0.05), likely due to the creation of melanoidins and the breakdown of proteins during this process [41]. Unlike GHES, MRPS revealed a decline in L* and a prominent rise in both a* and b* values (*p* < 0.05), confirming earlier published findings [40]. The primary factor behind the noted changes in the L*, a*, and b* values of MRPS is attributed to the generation of melanoidin throughout the MR process [12]. The c* value of GHES is significantly lower than that of the Control (*p* < 0.05), which is consistent with the findings reported in [42] that enzymatic digestion leads to a decrease in the c* value of Edible Bird’s Nest. The melanoidin during the MR process significantly increases the b* value of the samples [31]. According to the equation for c*, an increase in either a* and/or b* results in an increase in c*. Therefore, the c* value of MRPS is significantly higher than that of GHES (*p* < 0.05).

**Figure 2:**
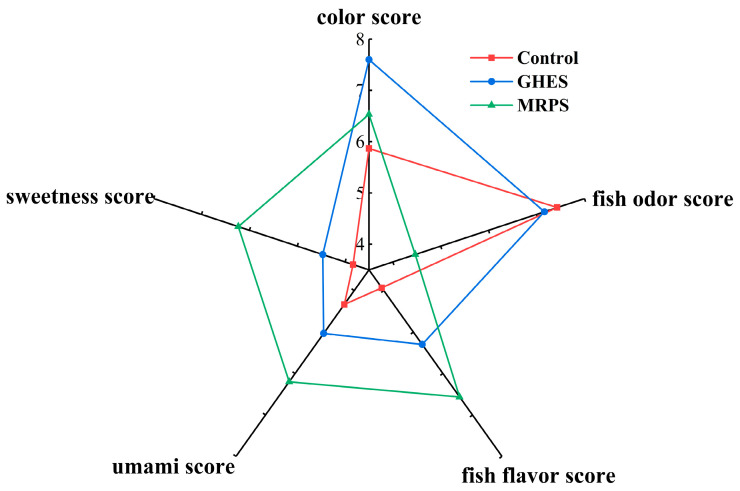
Sensory score radar chart of three different golden pomfret samples. Control: stewed golden pomfret samples; GHES: golden pomfret hydrolysate; MRPS: the products by facilitating the Maillard reaction (MR) between GHES and xylose. Higher scores for color, umami, fish flavor, and sweetness indicate good quality, while higher scores for fish odor indicate a heavy fish odor and poor quality.

This research examined five sensory characteristics to depict the flavor profile, comparing total flavor perception across three different sample groups, with the results of the sensory assessment shown in Figure 2. Notable differences in sensory scores were detected among these three groups (*p* < 0.05), suggesting that enzymatic digestion and the MR process significantly influenced the golden pomfret samples. The findings indicated that the GHES group displayed markedly higher levels of fish flavor, umami, sweetness, and color when contrasted with the Control group. This improvement is likely due to enzymatic hydrolysis, which aided in breaking down golden pomfret proteins, thus solubilizing amino acids associated with umami and sweetness (Glutamic acid, Aspartic acid, Glycine, Alanine, Threonine, Serine, and Proline), alongside flavor-boosting nucleotides, into the hydrolysates, ultimately enhancing the umami and sweetness of the sample [43]. Both the Control and GHES samples showed a more potent fish odor, whereas the MRPS group experienced a significant decrease in fish odor (*p* < 0.05). This decrease may result from volatile compounds generated by the MR process that conceal the fish odor, such as furans. Previous studies [44] have shown that substances like 2-Pentyl furan produce robust nutty, chocolate, and caramel scents that can mask fish odors. MRPS received the highest scores for fish flavor, umami, and sweetness, features resulting from the creation of intricate aromatic compounds and the enhancement of umami and richness in food products throughout the MR process [45]. Overall, the MR process contributed positively to the flavor profile and overall appeal of GHES.

### 3.4. Examination of Volatile Compounds

**Figure 3:**
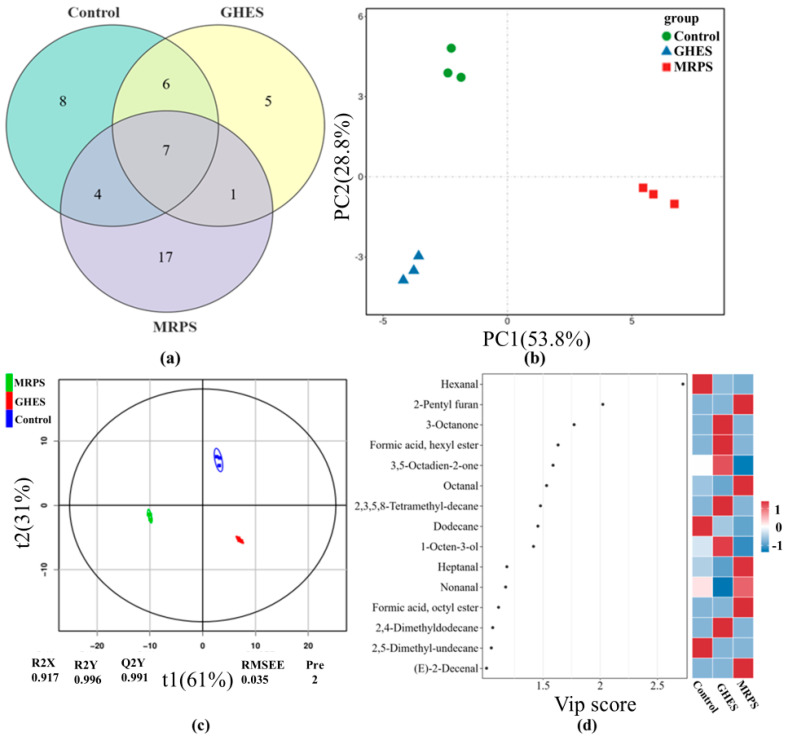
(**a**) Wayne’s Map; (**b**) PCA score plots; (**c**) OPLS-DA Score Chart; (**d**) VIP Score Chart of volatile compounds of three different golden pomfret samples. Control: stewed golden pomfret samples; GHES: golden pomfret hydrolysate; MRPS: the products by facilitating the Maillard reaction (MR) between GHES and xylose.

The diverse compositions of all samples are illustrated in Table 2. A total of 48 volatile substances were identified within the three sample categories, with the Control, GHES, and MRPS groups containing 25, 19, and 29 compounds, respectively. Seven compounds were found to be present in all three categories (Figure 3a), with the MRPS group exhibiting the most significant variety of volatile substances. The PCA scores of volatile compounds for the three sample categories are shown in Figure 3b, contributing to a cumulative variance of 82.6%, where PC1 represents 53.8%, and PC2 accounts for 28.8%. The Control, GHES, and MRPS groups were clearly distinguished, reflecting differences in flavor components among these groups (*p* < 0.05). In summary, the volatile compounds present in the three sample categories displayed both commonalities and distinctions.

MR generates various volatile compounds that contribute to the diversity of volatile compounds in the samples [46], which include all furans (like 2-Pentyl furan and 2-Octyl furan) and several aldehydes (such as 2-Nonenal, Decanal, (E)-2-Decenal, Undecanal, and 2-Undecenal) that are solely found in MR products (MRPS). Furans constitute a key category of volatiles generated during MR, primarily noted for their caramel and nut-like aromas [47,48]. Furthermore, furans are vital for augmenting the meat’s flavor, thus enhancing the overall taste profile [30]. The creation of 2-Pentyl furan and 2-Octyl furan adds delightful scents to MRPS, which include creamy, green bean, floral, and fruity undertones, further enriching the flavor of the samples. Aldehydes, being the main byproducts of lipid oxidation and amino acid breakdown, are recognized as significant compounds in food due to their unique flavors and low detection limits [49]. In the three groups studied, the proportions of aldehydes in relation to the total volatile compounds were as follows: Control (47.83 ± 4.86%), GHES (17.04 ± 1.48%), and MRPS (59.54 ± 1.58%). In both Control and GHES groups, Hexanal emerged as the leading aldehyde, while Octanal was the most prevalent aldehyde in the MRPS group. Hexanal is often linked with aquatic products, presenting a fish odor [50], whereas Octanal, which forms through the oxidation of oleic acid, offers a distinct herbal and oily fragrance [51].

Hydrocarbons arise from the auto-oxidation of long-chain fatty acids or from the thermo-oxidative breakdown of lipids [52]. Under particular conditions, these hydrocarbons can produce aldehydes and ketones, which could play a role in generating fish odors in shrimp paste [53]. The percentage of alkanes systematically declined from the Control group (28.29 ± 1.75%) to the MRPS group (0.37 ± 0.1%). This increase in aldehyde concentration, paired with the decline in alkane levels, may enhance the flavor profile of the sample [54]. Two alcohols, namely 1-Octen-3-ol and (E)-2-Undecen-1-ol, were detected in all three sample groups. The compound 1-Octen-3-ol, generated by lipoxygenase during the catalysis of unsaturated fatty acids, is a significant contributor to the fish odor [55] and accounted for 9.42 ± 1.93%, 15.87 ± 2.18%, and 5.71 ± 0.58% of the total volatile compounds in the three sample groups, respectively. After the application of MR treatment, a significant decrease in the relative amount of 1-Octen-3-ol was noted, which may improve the flavor profile of GHES samples by lessening the fish odor.

Ester compounds are important flavor agents because of their high volatility and comparatively low odor thresholds, which enable them to contribute fruity, sweet, and ester-like flavors to food products [56]. In the Control group, just a single ester component, Phthalic acid, di(2-propylpentyl) ester, was found. Conversely, the GHES group included Formic acid, hexyl ester. The MRPS group displayed a more intricate profile, comprising four ester compounds: Formic acid, heptyl ester; Formic acid, octyl ester; Carbonic acid, eicosyl vinyl ester; and 1,2-Benzenedicarboxylic acid, bis(2-methylpropyl) ester. After the MR treatment, there was a notable rise in both the number and variety of esters, which probably improved the desirable attributes of the samples, such as sweetness and ester aroma.

Ketones are recognized for releasing a pleasant fragrance at low concentrations; however, elevated concentrations can produce a sharp odor [57,58]. During the process of enzymatic hydrolysis, the share of ketones escalated from 9.00 ± 1.46% in the Control group to 26.67 ± 5.98% in the GHES group, before dropping to 3.83 ± 0.61% in the MRPS group after the MR. The relatively low levels of ketones in the MRPS group may assist in diminishing both pungent and fishy odors, considering that ketones can engage with other compounds in fish to amplify the perception of those odors [44]. Within the Control group, only palmitic acid was identified among the three sample groups, which might be due to acid loss during enzymatic digestion and MR.

To effectively pinpoint the volatile molecules that contribute to flavor differences among the three groups of samples, we utilized partial least squares discriminant analysis (PLS-DA) to analyze the volatile compounds. The explanatory power of the model is indicated by R^2^X and R^2^Y, while its ability to make predictions is illustrated by Q^2^; values of R^2^ and Q^2^ nearing 1.0 denote a high-quality model fit [59]. As shown in Figure 3c, we found R^2^Y = 0.996 and Q^2^Y = 0.991, suggesting that the model has significant explanatory and predictive strengths. The projected variable importance (VIP) values reflect the relevance of the components from each sample in the PLS-DA two-dimensional representation. A VIP score of ≥1 is viewed as a potential hallmark for distinguishing between samples, with higher VIP scores indicating more distinct differences. Figure 3d displays 15 volatile compounds that achieved VIP scores of ≥1: Hexanal, 2-Pentyl furan, 3-Octanone, Formic acid, hexyl ester, 3,5-Octadien-2-one, Octanal, 2,3,5,8-Tetramethylene-decane, Dodecane, 1-Octen-3-ol, Heptanal, Nonanal, Formic acid, octyl ester, 2,4-Dimethyldodecane, 2,5-Dimethyl-undecane, and (E)-2-Decenal. These volatile compounds could act as potential flavor indicators for differentiating among the distinct sample groups.

### 3.5. Examination of Distinctive Aroma Compounds

The influence of volatile compounds on the overall flavor profile of a food item is determined not only by their concentrations but also by their odor thresholds. Hence, it is vital to extract specific aromatic compounds from the identified volatiles to assess the effects of various treatments on a sample’s flavor. Relative Odor Activity Values (ROAVs) are often utilized to evaluate the role of volatile compounds in shaping the flavor profile of a food product. Compounds with ROAVs of ≥1 are regarded as key flavor compounds that substantially impact the overall flavor profile, while those with 0.1 ≤ ROAV < 1 are likely to influence the aroma. Table 3 lists seven key volatile compounds (ROAV ≥ 1) and three additional compounds that may affect the aroma (1 > ROAV ≥ 0.1). The key volatile compounds identified include 1-Octen-3-ol, Hexanal, Heptanal, Octanal, Nonanal, 2-Pentyl furan, and Decanal. The three compounds that could impact the aroma include Benzaldehyde, (E)-2-Octenal, and 2-Nonanone. According to the data in Table 3, major contributors to fish odor, specifically 1-Octen-3-ol and Hexanal, were found in both the Control and GHES groups. Their relative concentrations showed varying levels of reduction after MR treatment, indicating that the MR may help alleviate the fish odor in golden pomfret samples. Compared to the other two groups, the MRPS group displayed the highest concentrations of Heptanal (ROAV = 4.64), Octanal (ROAV = 7.89), and Decanal (ROAV = 6.14), possibly adding appealing aromas such as fatty, fruity, and floral notes to the samples [60]. Moreover, the main volatile compound in MRPS is 2-Pentyl furan (ROAV = 100), which imparts a fatty, fruity, and roasted flavor to the sample. In general, the MR treatment improved the distinctive aromatic components and intensified the flavor profile of the samples, while also decreasing the relative abundance of ichthyic compounds and improving the fish odor.

### 3.6. Correlation Analysis of Volatile Compounds and Sensory Descriptions

**Figure 4:**
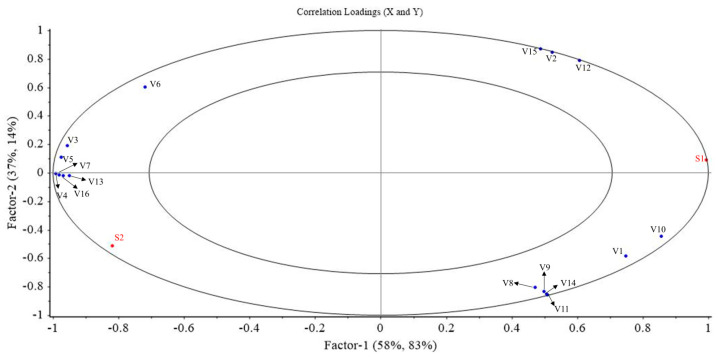
Partial least squares regression (PLSR) analysis of fish odor and fish flavor with some volatile compounds; sensory attributes are fish odor (S1) and fish flavor (S2). Volatile compounds including 1-Octen-3-ol (V1), Hexanal (V2), Heptanal (V3), 2-Pentyl furan (V4), Octanal (V5), Nonanal (V6), Decanal (V7), 3-Octanone (V8), Formic acid, hexyl ester (V9), 3,5-Octadien-2-one (V10), 2,3,5,8-Tetramethyl-decane (V11), Dodecane (V12), Formic acid, octyl ester (V13), 2,4-Dimethyldodecane (V14), 2,5-Dimethyl-undecane (V15), and (E)-2-Decenal (V16).

The X-axis was represented by the volatile compounds exhibiting an ROAV ≥ 1 and VIP ≥ 1, while the sensory parameters for fish odor (S1) and fish flavor (S2) constituted the Y-axis for constructing a partial least squares regression (PLSR) model. This model encompasses two components that explain 95% and 97% of the variance, respectively. The inner and outer ellipses denote 50% and 100% of the variance that has been clarified. The scores for sensory descriptions alongside the volatile compounds for the samples are found within these ellipses (R^2^ = 0.5 and 1.0), indicating a strong explanation by the PLSR model. As shown in Figure 4, the fish odor (S1) and certain volatile compounds, including 1-Octen-3-ol (V1), Hexanal (V2), 3-Octanone (V8), Formic acid, hexyl ester (V9), 3,5-Octadien-2-one (V10), 2,3,5,8-Tetramethyl-decane (V11), Dodecane (V12), 2,4-Dimethyldodecane (V14), and 2,5-Dimethyl-undecane (V15), are plotted on the right of the loadings graph, all falling outside the 50% explained variance. Conversely, the fish flavor (S2) along with volatile compounds such as Heptanal (V3), 2-Pentyl furan (V4), Octanal (V5), Nonanal (V6), Decanal (V7), Formic acid, octyl ester (V13), and (E)-2-Decenal (V16) occupy the left side. These findings suggest a primary association of fish odor with volatile compounds like 1-Octen-3-ol (V1) and Hexanal (V2), while fish flavor is mainly correlated with components such as 2-Pentyl furan (V4).

### 3.7. Analysis of Free Amino Acids, 5′-Nucleotides, and Organic Acids

The flavor profile of aquatic products is significantly influenced by the composition and concentration of free amino acids. By enhancing the release of these amino acids, both the flavor and nutritional quality of food products can be improved. Flavored protease, which is a non-specific type of protease, comprises a blend of endopeptidases and exopeptidases aimed at the N-terminus of proteins, hydrolyzing peptide bonds to liberate amino acids [61,62]. Enzymatic hydrolysis of protein from golden pomfret yields taste-active compounds, such as free amino acids (FAAs) and low-molecular-weight peptides known for their distinct flavors. These compounds play a vital role not only as important flavor enhancers in culinary applications but also as substrates for the MR, promoting the formation of additional flavor components [63]. Table 4 depicts the distribution of FAAs among three sample categories, with measured total FAA contents of the Control (56.78 ± 2.70 mg/100 g), GHES (60.56 ± 0.76 mg/100 g), and MRPS (76.83 ± 1.78 mg/100 g), respectively. The continuous rise in total FAAs throughout the reaction signifies that both the MR and enzymatic hydrolysis promote the breakdown of proteins and peptides into FAAs. Fu et al. found that, compared with traditional stewing methods, enzymatic hydrolysis can more effectively promote the release of amino acids from tilapia heads into the hydrolysate [7]. The levels of FAAs during the MR are affected by both their production and consumption rates. The thermal degradation of proteins and peptides produces FAAs, while carbonyl compounds present in the MR result in their utilization [64,65]. Importantly, the FAA concentration in MRPS was considerably greater than that in GHES (*p* < 0.05), indicating that the FAA production during the MR was higher than the consumption rate. A total of 16 amino acids were detected in the three sample groups, which is consistent with the results detected by Qiu et al. [66] in the fermented golden pomfret samples. Among them, sweet amino acids (Glycine, Alanine, Threonine, Serine, Proline) were dominant, followed by bitter amino acids (Histidine, Arginine, Valine, Methionine, Isoleucine, Leucine, Phenylalanine, Tryptophan, Lysine) and umami amino acids (Glutamic acid, Aspartic acid). Glutamic acid and Aspartic acid are well-known for being typical amino acids that provide umami, resembling the taste of monosodium glutamate (MSG); they not only enhance the umami but also work synergistically with 5′-nucleotides to significantly boost the umami flavor in food products [67]. Overall, the samples demonstrated a marked increase (*p* < 0.05) in the concentrations of sweet and umami amino acids, including Glycine, Alanine, Glutamic acid, and Aspartic acid, after enzymatic digestion and MR, thus contributing to the improved sweetness and umami of the sample.

Nitrogenous compounds known as free nucleotides are vital for influencing the flavor profiles of aquatic products, with specific 5′-nucleotides playing a crucial role in the umami taste [68,69]. This research focused on assessing the varying levels of five key flavor nucleotides: 5′-GMP, 5′-IMP, 5′-AMP, 5′-CMP, and 5′-UMP. Throughout the reaction, the total concentration of 5′-nucleotides (Table 4) showed a steady increase, reaching its peak in the 5′-nucleotides of MRPS (63.45 ± 0.32 mg/100 g), followed by GHES (26.01 ± 7.51 mg/100 g), while the Control group had the lowest concentration (13.65 ± 0.08 mg/100 g). Table 4 depicts the levels of 5′-nucleotides among the three sample groups, revealing that GMP and AMP were ranked as MRPS > GHES > Control. This trend is likely due to the enzyme aiding the transfer of 5′-nucleotides from the golden pomfret into the hydrolysates, with heating conditions further promoting this transfer [70]. Moreover, it is crucial to take into account the thermal degradation of 5′-nucleotides during the heating process, as the MRPS might show decreased amounts of certain nucleotides, such as 5′-UMP, 5′-CMP, and 5′-IMP [71,72]. Following the process of the MR, the levels of 5′-AMP and 5′-GMP were found to rise, which is likely attributable to the degradation rate of heat being slower than the migration of raw material into the hydrolysates. In summary, both enzymatic digestion and MR significantly elevated (*p* < 0.05) the concentration of 5′-nucleotides in the samples, notably GMP, thereby augmenting the umami flavor in the sample.

Nucleotides and amino acids that produce umami taste can act together to boost umami flavor, which is generally assessed through equivalent umami concentration (EUC). The levels of EUC show a positive correlation with the quantities of MSG analogs [73]. Among the three groups studied, the MRPS group demonstrated the highest EUC value at 1.2 ± 0.03 g/100 g, whereas the GHES group followed with a value of 0.18 ± 0.11 g/100 g, and the Control group had the lowest measurement at 0.02 ± 0.00 g/100 g (Table 4). These findings suggest that both enzymatic hydrolysis and the MR significantly enhanced the umami flavor of golden pomfret samples (*p* < 0.05).

Organic acids are crucial for maintaining the nutritional quality and value of food items [74]. The variety and amount of organic acids, a key group of acidic components, have a direct influence on the flavor profile of seafood products [75]. As noted in earlier studies [76], lactic acid not only improves flavor but also increases buffering capacity, while succinic acid and its sodium counterpart exhibit umami flavor qualities and properties that enhance umami similar to those of glutamic acid. In Table 4, the detection of four different acids—lactic, malic, citric, and succinic—is illustrated across three sets of samples. The generation of lactic acid is mainly linked to the anaerobic decomposition of glycogen in animal bodies after slaughter [77]. The GHES group demonstrated a lower production compared to the Control group, likely as a result of the creation of alkaline groups throughout the enzymatic activity, which contributed to a reduction in lactic acid levels. In contrast, fish soup samples post-MR exhibited a marked increase (*p* < 0.05) in lactic acid concentration, likely due to the reduction of alkaline groups that promote lactic acid production during the MR process [78]. The heightened lactic acid levels in the MRPS group may have contributed to an intensified umami flavor in the sample. Previous studies [79] indicate that lactic acid can serve as a replacement for some monosodium glutamate (MSG), while functioning as a flavor enhancer. Notably, the Control group had a substantially greater concentration of citric acid in comparison to the other two groups. Research has shown that elevated citric acid levels can significantly lessen the perception of umami intensity [80]. As a result, a decrease in citric acid levels might improve the awareness of umami flavor in the samples. Organic acids play a crucial role in the synthesis and metabolism of aromatic compounds, esters, and amino acids. The pronounced decline (*p* < 0.05) in citric acid concentrations in the GHES and MRPS groups can be linked to decarboxylation processes that take place during enzymatic and MR treatments [81]. No noteworthy differences in concentrations of malic and succinic acid were observed among the three samples (*p* < 0.05).

### 3.8. Antioxidant Activity

The MR not only enhances the unique aroma and taste of food products but also boosts their antioxidant properties [82]. Recent research has explored the antioxidant potential of protein hydrolysates and compounds derived from marine sources located in aquatic environments [83,84,85]. Table 5 illustrates the outcomes of the antioxidant activities. The samples of Control, GHES, and MRPS exhibited the capacity to scavenge DPPH, ABTS, and hydroxyl radicals, with the effectiveness of scavenging escalating with increasing concentrations, ranked as MRPS > GHES > Control. As the concentration progressed from 10 mg/mL to 50 mg/mL, the DPPH clearance for MRPS, GHES, and Control increased from 72.23 ± 1.58%, 63.73 ± 0.51%, and 61.93 ± 0.87% to 83.47 ± 0.35%, 81.53 ± 3.18%, and 80.23 ± 1.3%, respectively (*p* < 0.05). These values were higher than those reported by [86] in the hydrolysates of bighead carp. The ABTS scavenging improved from 74.03 ± 2.19%, 66.9 ± 0.17%, and 60.77 ± 1.37% to 87.23 ± 2.65%, 85.77 ± 4.32%, and 81.13 ± 1.95%, respectively (*p* < 0.05). Additionally, scavenging of hydroxyl radicals increased from 88.73 ± 2.51%, 79.4 ± 1.28%, and 72.8 ± 2.52% to 98.1 ± 0.36%, 94.77 ± 0.42%, and 94.43 ± 0.7%, respectively (*p* < 0.05). The ferric ion reduction capability did not exhibit a significant difference between the Control and GHES groups (*p* < 0.05). Nevertheless, MRPS demonstrated a significantly higher ferric ion reduction capacity when compared to the previous two groups (*p* < 0.05). GHES demonstrated a notable increase in ABTS and hydroxyl radical scavenging activities compared to the Control group (*p* < 0.05). This improvement is likely a result of enzymatic hydrolysis that liberates antioxidant peptides, thus boosting free radical scavenging capabilities [87]. Compared to the first two groups, MRPS indicated a significant enhancement in scavenging activities for DPPH, ABTS, hydroxyl radicals, as well as iron ion reduction (*p* < 0.05). This enhancement can be attributed to the formation of melanoidins during the MR process [88]. These melanoidins operate through various mechanisms, including chain-breaking, oxygen scavenging, and chelation of metals [89].

## 4. Conclusions

This study aims to enhance the flavor profile of golden pomfret products through the MR between GHES with reducing sugars. Spectroscopic investigations revealed that enzymatic digestion modified the structure of golden pomfret proteins, while the MR facilitated the formation of intermediates and melanoidins in MRPS. Sensory evaluation results demonstrated that both enzymatic and MR treatments improved fish flavor, sweetness, and umami, while simultaneously reducing the fish odor. Analysis of flavor compounds indicated that MR treatment increased the production of volatile molecules in GHES, while decreasing the relative concentration of fish odor compounds. Furthermore, enzymatic digestion and MR enhanced the concentrations of compounds such as free amino acids and 5′-nucleotides, which significantly elevated the umami flavor of samples (*p* < 0.05). In vitro antioxidant assays indicated that enzymatic digestion and MR significantly improved the DPPH, ABTS, hydroxyl radical scavenging ability, and ferric ion reduction capacity of golden pomfret samples (*p* < 0.05). In conclusion, enzymatic digestion and the MR not only enhanced the flavor but also increased the antioxidant activity of golden pomfret. Future research will focus on identifying peptides that enhance flavor and elucidating their impact on the taste of golden pomfret. This study elucidated the role of the MR in improving the flavor of golden pomfret hydrolysate, thus contributing to the development of the golden pomfret processing industry.

## Figures and Tables

**Table 1:** Color of three different golden pomfret samples.

Sample	L*	a*	b*	c*
Control	33.98 ± 0.14 ^c^	−1.17 ± 0.04 ^b^	−2.42 ± 0.09 ^c^	2.69 ± 0.06 ^b^
GHES	38.82 ± 0.13 ^a^	−1.71 ± 0.03 ^c^	−0.4 ± 0.11 ^b^	1.76 ± 0.02 ^c^
MRPS	35.93 ± 0.17 ^b^	−1.03 ± 0.03 ^a^	3.05 ± 0.05 ^a^	3.22 ± 0.05 ^a^

Different letters mean the significance (*p* < 0.05). Control: stewed golden pomfret samples; GHES: golden pomfret hydrolysate; MRPS: the products by facilitating the Maillard reaction (MR) between GHES and xylose.

**Table 2:** Relative content of volatile compounds (%) of three different golden pomfret samples.

Volatile Compounds	Relative Content (%)		
	Control	GHES	MRPS
Hexanal	31.15 ± 2.75 ^a^	11.52 ± 0.7 ^b^	10.46 ± 0.83 ^b^
Heptanal	1.62 ± 1.41 ^b^	-	7.78 ± 0.73 ^a^
Benzaldehyde	1.62 ± 0.24 ^a^	-	2.51 ± 0.8 ^a^
Octanal	1.6 ± 0.1 ^b^	-	12.45 ± 2.47 ^a^
(E)-2-Octenal	-	-	2.4 ± 0.51 ^a^
Nonanal	9.67 ± 1.07 ^a^	5.08 ± 0.75 ^b^	12.14 ± 2.22 ^a^
(E)-2-Nonenal	-	-	1.96 ± 0.79 ^a^
Decanal	-	-	1.03 ± 0.23 ^a^
2,4-Dimethyl-benzaldehyde	2.17 ± 0.5 ^a^	0.44 ± 0.03 ^b^	-
(E, E)-2,4-Nonadienal	-	-	0.09 ± 0.04 ^a^
1,2-Dimethyl-4-oxocyclohex-2-enecarboxaldehyde	-	-	0.57 ± 0.23 ^a^
(E)-2-Decenal	-	-	4.95 ± 1.3 ^a^
Undecanal	-	-	0.28 ± 0.09 ^a^
2-Undecenal	-	-	2.9 ± 1 ^a^
Aldehydes	47.83 ± 4.86 ^b^	17.04 ± 1.48 ^c^	59.54 ± 1.58 ^a^
1-Octen-3-ol	9.42 ± 1.93 ^b^	15.87 ± 2.18 ^a^	5.71 ± 0.58 ^c^
trans-2-Undecen-1-ol	-	2.92 ± 0.61 ^a^	-
Alcohols	9.42 ± 1.93 ^b^	18.79 ± 2.78 ^a^	5.71 ± 0.58 ^b^
Formic acid, hexyl ester	-	9.04 ± 2.21 ^a^	-
Formic acid, heptyl ester	-	-	2.81 ± 0.88 ^a^
Formic acid, octyl ester	-	-	6.11 ± 1.87 ^a^
Carbonic acid, eicosyl vinyl ester	-	-	0.03 ± 0.02 ^a^
1,2-Benzenedicarboxylic acid, bis(2-methylpropyl) ester	-	-	0.03 ± 0.02 ^a^
Phthalic acid, di(2-propylpentyl) ester	4.11 ± 3.72 ^a^	-	-
Esters	4.11 ± 3.72 ^b^	9.04 ± 2.21 ^a^	8.97 ± 2.8 ^a^
3-Octanone	-	11.07 ± 4.22 ^a^	-
3,5-Octadien-2-one	9 ± 1.46 ^b^	15.6 ± 3.3 ^a^	1.36 ± 1.18 ^c^
2-Nonanone	-	-	0.38 ± 0.28 ^a^
3-Nonen-2-one	-	-	0.37 ± 0.32 ^a^
2-Decanone	-	-	1.72 ± 0.17 ^a^
Ketones	9.00 ± 1.46 ^b^	26.67 ± 5.98 ^a^	3.83 ± 0.61 ^b^
2,3,5,8-Tetramethyl-decane	-	7.21 ± 0.28 ^a^	-
Dodecane	6.38 ± 0.53 ^a^	0.99 ± 0.29 ^b^	-
2,5-Dimethyl-undecane	2.94 ± 0.32 ^a^	-	-
4-Methyl-dodecane	2.87 ± 0.33 ^a^	1.86 ± 0.47 ^b^	-
4,6-Dimethyl-dodecane	1.52 ± 0.16 ^a^	1.7 ± 0.6 ^a^	-
2,4-Dimethyldodecane	-	3.73 ± 0.35 ^a^	-
5-ethyl-5-methyl-Decane	1.63 ± 0.26 ^a^	1.31 ± 0.21 ^a^	-
2,6,10-Trimethyltridecane	1.23 ± 0.12 ^a^	-	-
Tetradecane	3.19 ± 0.59 ^a^	1.88 ± 0.54 ^b^	0.15 ± 0.07 ^c^
2,6,11,15-Tetramethyl-hexadecane	1.15 ± 0.19 ^a^	-	-
Pentadecane	3.63 ± 0.54 ^b^	4.49 ± 0.08 ^a^	0.17 ± 0.08 ^c^
Hexadecane	0.55 ± 0.07 ^a^	-	0.02 ± 0.01 ^b^
Heptadecane	1.25 ± 0.25 ^b^	1.86 ± 0.14 ^a^	-
2,6,10,14-Tetramethyl-pentadecane	0.81 ± 0.04 ^b^	1.09 ± 0.21 ^a^	0.03 ± 0.02 ^c^
3-Methyl-5-propyl-nonane	1.13 ± 0.27 ^a^	-	-
Alkanes	28.29 ± 1.75 ^a^	26.12 ± 1.3 ^a^	0.37 ± 0.1 ^b^
2-Pentyl furan	-	-	19.47 ± 2.39 ^a^
2-n-Octyl furan	-	-	2 ± 0.74 ^a^
Furans	-	-	21.47 ± 2 ^a^
n-Hexadecanoic acid	1.03 ± 0.87 ^a^	-	-
Acids	1.03 ± 0.87 ^a^	-	-
Butylated Hydroxytoluene	-	2.34 ± 0.19 ^a^	0.11 ± 0.04 ^b^
(Z)-9-Octadecenamide	0.25 ± 0.1 ^a^	-	-
Octadecanamide	0.08 ± 0.05 ^a^	-	-
Others	0.32 ± 0.11 ^b^	2.34 ± 0.19 ^a^	0.11± 0.04 ^b^

“-” represents that not detected. Different letters mean the significance (*p* < 0.05). Control: stewed golden pomfret samples; GHES: golden pomfret hydrolysate; MRPS: the products by facilitating the Maillard reaction (MR) between GHES and xylose.

**Table 3:** Relative odor activity values (ROAVs) of the volatile compounds of three different golden pomfret samples.

Compound	Molecular	Threshold	ROAV		
	Formula	(mg/kg)	Control	GHES	MRPS
1-Octen-3-ol	C_8_H_16_O	0.01	100	100	17.01
Hexanal	C_6_H_12_O	0.21	15.75	3.45	1.48
Heptanal	C_7_H_14_O	0.05	3.44	-	4.64
Benzaldehyde	C_7_H_6_O		0.57	-	0.25
Octanal	C_8_H_16_O	0.047	3.61	-	7.89
Nonanal	C_9_H_18_O	0.045	22.81	7.1	8.04
2-Pentyl furan	C_9_H_14_O	0.0058	-	-	100
(E)-2-Octenal	C_8_H_14_O	0.083	-	-	0.86
Decanal	C_10_H_20_O	0.005	-	-	6.14
2-Nonanone	C_9_H_18_O	0.05	-	-	0.23

Threshold of volatile compounds was obtained from a book titled *Compilations of Odor Threshold Values in Air, Water and Other Media* (second enlarged and revised edition). “-” represents that not detected. Control: stewed golden pomfret samples; GHES: golden pomfret hydrolysate; MRPS: the products by facilitating the Maillard reaction (MR) between GHES and xylose.

**Table 4:** The content of free amino acids, 5′-nucleotides, and organic acids (mg/100 g) of three different golden pomfret samples.

Non-Volatile Compounds	Content (mg/100 g)		
	Control	GHES	MRPS
Aspartic acid	0.55 ± 0.01 ^c^	1.16 ± 0.01 ^b^	1.46 ± 0.05 ^a^
Glutamic acid	3.22 ± 0.01 ^c^	6.84 ± 0.08 ^b^	8.16 ± 0.18 ^a^
Umami amino acid	3.77 ± 0.00 ^c^	8.00 ± 0.08 ^b^	9.61 ± 0.22 ^a^
Serine	1.39 ± 0.05 ^b^	1.45 ± 0.01 ^b^	1.71 ± 0.04 ^a^
Glycine	34.08 ± 0.17 ^b^	29.63 ± 0.48 ^c^	35.81 ± 0.76 ^a^
Threonine	1.89 ± 0.18 ^c^	2.15 ± 0.03 ^b^	2.90 ± 0.05 ^a^
Alanine	4.60 ± 0.48 ^c^	6.57 ± 0.11 ^b^	8.35 ± 0.17 ^a^
Proline	0.33 ± 0.07 ^b^	0.52 ± 0.02 ^b^	3.02 ± 0.89 ^a^
Sweet amino acid	42.29 ± 0.85 ^b^	40.32 ± 0.60 ^c^	51.80 ± 1.27 ^a^
Histidine	1.17 ± 0.37 ^a^	1.34 ± 0.06 ^a^	1.07 ± 0.02 ^a^
Arginine	3.26 ± 1.27 ^a^	3.52 ± 0.07 ^a^	3.98 ± 0.10 ^a^
Valine	0.35 ± 0.01 ^b^	-	0.86 ± 0.04 ^a^
Methionine	0.69 ± 0.09 ^b^	0.79 ± 0.16 ^ab^	1.01 ± 0.03 ^a^
Isoleucine	0.84 ± 0.03 ^b^	-	1.02 ± 0.03 ^a^
Leucine	0.95 ± 0.12 ^b^	-	1.36 ± 0.09 ^a^
Phenylalanine	0.36 ± 0.16 ^b^	0.54 ± 0.07 ^b^	0.86 ± 0.12 ^a^
Tryptophan	0.55 ± 0.23 ^c^	2.68 ± 0.10 ^a^	1.48 ± 0.08 ^b^
Lysine	2.54 ± 0.05 ^c^	3.37 ± 0.09 ^b^	3.78 ± 0.05 ^a^
Bitter amino acid	10.72 ± 2.01 ^b^	12.25 ± 0.15 ^b^	15.42 ± 0.46 ^a^
Total free amino acid	56.78 ± 2.70 ^c^	60.56 ± 0.76 ^b^	76.83 ± 1.78 ^a^
5′-CMP	8.06 ± 0.05 ^a^	5.39 ± 2.08 ^b^	1.29 ± 0.01 ^c^
5′-UMP	0.53 ± 0.00 ^b^	1.4 ± 0.02 ^a^	0.55 ± 0.01 ^b^
5′-GMP	-	6.82 ± 5.91 ^b^	49.67 ± 0.25 ^a^
5′-IMP	2.81 ± 0.02 ^a^	2.79 ± 0.52 ^a^	1.97 ± 0.01 ^b^
5′-AMP	2.25 ± 0.04 ^c^	9.59 ± 0.04 ^b^	9.97 ± 0.06 ^a^
Total 5′-nucleotides	13.65 ± 0.08 ^c^	26.01 ± 7.51 ^b^	63.45 ± 0.32 ^a^
Lactic acid	150.42 ± 3.28 ^b^	139.8 ± 2.55 ^c^	161.44 ± 17.66 ^a^
Malic acid	9.20 ± 0.32 ^a^	9.33 ± 0.91 ^a^	9.80 ± 0.87 ^a^
Citric acid	105.16 ± 3.53 ^a^	53.01 ± 3.18 ^b^	53.18 ± 1.01 ^b^
Succinic acid	2.54 ± 0.46 ^a^	2.12 ± 0.02 ^a^	2.15 ± 0.12 ^a^
Total organic acids	267.32 ± 5.87 ^a^	204.26 ± 5.62 ^b^	226.57 ± 17.99 ^b^
EUC	0.02 ± 0.00 ^c^	0.18 ± 0.11 ^b^	1.20 ± 0.03 ^a^

“-” represents that not detected. Different letters mean the significance (*p* < 0.05). The unit of EUC is g/100 g. Control: stewed golden pomfret samples; GHES: golden pomfret hydrolysate; MRPS: the products by facilitating the Maillard reaction (MR) between GHES and xylose. The table expresses the content of non-volatile compounds contained in 100 g of the supernatant obtained after centrifugation.

**Table 5:** Antioxidant capacity (%) of three different golden pomfret samples.

Sample	Antioxidant Capacity (%)				
	Concentration (mg/mL)	Control	GHES	MRPS	Vc
DPPH	10	61.93 ± 0.87 ^Ce^	63.73 ± 0.51 ^Cc^	72.23 ± 1.58 ^Bd^	98.53 ± 0.93 ^Aa^
	20	64.27 ± 0.57 ^Cd^	65.67 ± 1.45 ^Cc^	73.90 ± 1.05 ^Bcd^	98.87 ± 0.40 ^Aa^
	30	70.63 ± 0.67 ^Cc^	71.90 ± 1.39 ^Cb^	75.63 ± 2.01 ^Bbc^	98.5 ± 0.98 ^Aa^
	40	72.93 ± 0.76 ^Cb^	73.17 ± 0.40 ^Cb^	77.40 ± 1.21 ^Bb^	99.23 ± 0.40 ^Aa^
	50	80.23 ± 1.30 ^Ba^	81.53 ± 3.18 ^Ba^	83.47 ± 0.35 ^Ba^	98.93 ± 0.81 ^Aa^
ABTS	10	60.77 ± 1.37 ^De^	66.90 ± 0.17 ^Cc^	74.03 ± 2.19 ^Bb^	98.37 ± 0.51 ^Aa^
	20	64.23 ± 0.51 ^Dd^	70.80 ± 3.52 ^Cbc^	75.90 ± 1.15 ^Bb^	97.63 ± 1.96 ^Aa^
	30	73.47 ± 1.39 ^Cc^	75.10 ± 2.25 ^BCb^	77.47 ± 1.96 ^Bb^	97.9 ± 0.72 ^Aa^
	40	77.27 ± 1.37 ^Cb^	83.97 ± 1.81 ^Ba^	85.53 ± 2.81 ^Ba^	98.97 ± 1.04 ^Aa^
	50	81.13 ± 1.95 ^Ca^	85.77 ± 4.32 ^BCa^	87.23 ± 2.65 ^Ba^	99.47 ± 0.29 ^Aa^
Hydroxyl radical	10	72.80 ± 2.52 ^Dd^	79.40 ± 1.28 ^Cd^	88.73 ± 2.51 ^Bc^	95.5 ± 0.72 ^Ab^
	20	80.67 ± 2.72 ^Cc^	85.27 ± 3.06 ^Bc^	91.47 ± 1.80 ^Abc^	95.9 ± 0.72 ^Ab^
	30	85.77 ± 1.94 ^Cbc^	87.80 ± 2.14 ^Bbc^	90.93 ± 3.21 ^ABbc^	95.03 ± 0.70 ^Ab^
	40	90.00 ± 4.11 ^Bab^	90.67 ± 3.35 ^ABab^	94.27 ± 1.56 ^ABab^	96.23 ± 0.61 ^Ab^
	50	94.43 ± 0.70 ^Ba^	94.77 ± 0.42 ^Ba^	98.10 ± 0.36 ^Aa^	98.17 ± 0.97 ^Aa^
Ferric ion reduction	10	0.44 ± 0.00 ^Cd^	0.45 ± 0.01 ^Cc^	0.50 ± 0.01 ^Bd^	2.84 ± 0.00 ^Ab^
	20	0.45 ± 0.00 ^Ccd^	0.46 ± 0.01 ^Cbc^	0.54 ± 0.02 ^Bc^	2.85 ± 0.00 ^Aa^
	30	0.45 ± 0.01 ^Cc^	0.47 ± 0.01 ^Cb^	0.59 ± 0.01 ^Bb^	2.85 ± 0.00 ^Aa^
	40	0.47 ± 0.00 ^Cb^	0.48 ± 0.01 ^Cb^	0.63 ± 0.01 ^Ba^	2.85 ± 0.00 ^Aa^
	50	0.50 ± 0.01 ^Da^	0.52 ± 0.01 ^Ca^	0.65 ± 0.01 ^Ba^	2.85 ± 0.00 ^Aa^

Different letters mean the significance (*p* < 0.05); upper case letters indicate significance between Control, GHES, MRPS, and Vc; lower case letters indicate significance between different concentrations of the same sample. Control: stewed golden pomfret samples; GHES: golden pomfret hydrolysate; MRPS: the products by facilitating the Maillard reaction (MR) between GHES and xylose; Vc: L-Ascorbic acid. The concentrations ranging from 10 to 50 mg/mL were obtained by diluting the supernatants of the three groups of samples with distilled water.

## Data Availability

The original contributions presented in this study are included in the article/Supplementary Materials. Further inquiries can be directed to the corresponding author.

## Supplementary Materials

The following supporting information can be downloaded at https://www.mdpi.com/article/10.3390/foods14040560/s1: Table S1: Optimisation of hydrolysate conditions(Including enzyme type, enzyme digestion time, enzyme digestion temperature, pH, enzyme additions) for golden pomfret; Table S2: Optimisation of the MR conditions(Including reaction temperature, reaction time, pH and xylose additions) between gold pomfret hydrolysate (GHES) and xylose.

## Abbreviations

The following abbreviations are used in this manuscript:

MR	Maillard reaction
GHES	Gold pomfret hydrolysate
EUC	Equivalent umami concentration
ROAV	Relative odor activity value
