# Supplementary material for: Combining Sensory Analysis and Flavoromics to Determine How the Maillard Reaction Affects the Flavors of Golden Pomfret Hydrolysates
Source: Foods. 2025 Feb 8;14(4):560. doi: 10.3390/foods14040560 (PMC11854427; doi:10.3390/foods14040560)
Supplement: Supplementary file 1 [file foods-14-00560-s001.zip › foods-3413939-supplementary.pdf]

Table S1: Optimisation of hydrolysate conditions(Including enzyme type, enzyme digestion time, enzyme digestion temperature, pH, enzyme additions) for golden pomfret.

**Enzymatic conditions**		**DH (%)**				
		**Flavored protease**	**Neutral protease**	**Bromelain**	**Papain**	**Complex protease**
50℃, 4h, 0.8%	pH6.0	13.28±0.5^{Ad}	11.83±0.45^{Bc}	9.88±0.6^{Cd}	13.58±0.15^{Ae}	11.6±0.21^{Bd}
	pH6.5	16.85±0.55^{Ab}	13.42±0.56^{Cb}	12.29±0.35^{Dc}	15.6±0.35^{Bc}	13.65±0.2^{Cb}
	pH7.0	23.3±0.41^{Aa}	16.39±0.4^{Ca}	16.23±0.6^{Ca}	17.95±0.25^{Ba}	18.38±0.15^{Ba}
	pH7.5	15.76±0.5^{Ac}	14.18±0.4^{Bb}	13.68±0.51^{Bb}	16.26±0.15^{Ab}	13.71±0.25^{Bb}
	pH8.0	14.21±0.45^{Ad}	12.26±0.55^{Bc}	9.38±0.72^{Cd}	14.41±0.25^{Ad}	12.75±0.26^{Bc}
4h, 0.8%, ph7	40℃	15.6±0.4^{Ad}	8.92±0.6^{Cd}	7.69±0.6^{De}	13.42±0.25^{Be}	13.55±0.26^{Bd}
	45℃	16.69±0.94^{Acd}	14.9±0.45^{Bc}	10.94±0.35^{Cd}	15.43±0.2^{Bc}	15.47±0.25^{Bb}
	50℃	23.2±0.56^{Aa}	16.52±0.5^{Cb}	16.13±0.55^{Ca}	18.57±0.25^{Ba}	18.11±0.26^{Ba}
	55℃	20.26±0.3^{Ab}	17.75±0.47^{Ba}	14.27±0.5^{Db}	16.46±0.21^{Cb}	14.61±0.25^{Dc}
	60℃	18.18±1.52^{Ac}	16.16±0.4^{Bb}	12.29±0.65^{Dc}	14.41±0.15^{Cd}	13.75±0.2^{CDd}
50℃, 4h, ph7	0.20%	11.86±0.6^{Bd}	9.71±0.45^{Cc}	7.56±0.5^{Dd}	14.54±0.36^{Ac}	8.36±0.38^{Dd}
	0.40%	16.69±0.74^{Ac}	11.96±0.3^{Cb}	11.96±0.36^{Cc}	14.7±0.3^{Bc}	14.74±0.2^{Bc}
	0.60%	18.04±0.51^{Ab}	12.85±0.81^{Db}	14.31±0.55^{Cb}	15.66±0.15^{Bb}	16.62±0.36^{Bb}
	0.80%	22.81±0.4^{Aa}	16.85±0.35^{Ca}	16.32±0.65^{Ca}	17.98±0.41^{Ba}	18.47±0.25^{Ba}
	1.00%	22.48±0.75^{Aa}	17.65±1.05^{BCa}	16.59±0.94^{Ca}	18.18±0.55^{Ba}	18.74±0.25^{Ba}
50℃, 0.8%, ph7	2h	19.83±0.7^{Ad}	13.32±0.38^{Dd}	13.38±0.45^{Dd}	16.79±0.25^{Cd}	18.34±0.15^{Bd}
	4h	23.14±0.8^{Ac}	16.32±0.36^{Cc}	15.66±0.45^{Cc}	18.57±0.35^{Bc}	19.33±0.32^{Bc}
	6h	26.71±0.49^{Ab}	17.38±0.32^{Cb}	16.69±0.49^{Cb}	19.5±0.36^{Bb}	19.83±0.15^{Bb}
	8h	28.36±0.47^{Aa}	17.95±0.41^{Cab}	20.33±0.49^{Ba}	20.39±0.26^{Ba}	20.33±0.21^{Ba}
	10h	29.25±0.41^{Aa}	18.61±0.45^{Ca}	20.86±0.35^{Ba}	20.72±0.15^{Ba}	20.72±0.25^{Ba}

Different letters mean the significance ($p < 0.05$).

Table S2: Optimisation of the MR conditions(Including reaction temperature, reaction time, pH and xylose additions) between gold pomfret hydrolysate (GHES) and xylose.

**Reaction conditions**		**A_{294}**	**A_{420}**	**Senses Score**
1.5h, 8%, 120℃	pH5	0.376±0.002^{d}	0.062±0.002^{d}	3.8±1.23^{b}
	pH6	0.607±0.005^{c}	0.177±0.001^{c}	4.5±1.18^{b}
	pH7	0.672±0.007^{a}	0.189±0.002^{c}	5.9±0.74^{a}
	pH8	0.679±0.005^{a}	0.209±0.014^{b}	4.2±0.79^{b}
	pH9	0.653±0.008^{b}	0.234±0.01^{a}	3.4±1.07^{b}
pH7, 8%, 120℃	0.5h	0.539±0.007^{c}	0.177±0.002^{e}	3.5±1.08^{c}
	1.0h	0.541±0.003^{c}	0.189±0.007^{d}	4.3±1.06^{bc}
	1.5h	0.673±0.003^{b}	0.204±0.007^{c}	5.3±0.67^{b}
	2.0h	0.776±0.017^{a}	0.232±0.007^{b}	6.9±0.74^{a}
	2.5h	0.788±0.002^{a}	0.253±0.002^{a}	4.5±1.35^{bc}
pH7, 8%, 2.0h	100℃	0.556±0.002^{d}	0.184±0.002^{d}	4.6±0.7^{b}
	110℃	0.615±0.008^{c}	0.2±0.009^{c}	5.3±1.06^{b}
	120℃	0.774±0.007^{a}	0.224±0.008^{b}	6.7±0.67^{a}
	130℃	0.694±0.003^{b}	0.233±0.002^{b}	4.7±0.95^{b}
	140℃	0.684±0.01^{b}	0.246±0.002^{a}	4.3±1.06^{b}
pH7, 2.0h, 120℃	2.0%	0.583±0.007^{c}	0.166±0.003^{d}	4.5±0.85^{c}
	4.0%	0.586±0.009^{c}	0.173±0.003^{d}	4.9±1.2^{bc}
	6.0%	0.613±0.005^{c}	0.195±0.002^{c}	5.6±0.52^{b}
	8.0%	0.754±0.016^{b}	0.219±0.004^{b}	6.9±0.88^{a}
	10.0%	0.818±0.054^{a}	0.236±0.006^{a}	5.6±0.97^{b}

Different letters mean the significance ($p < 0.05$).The values of A294 and A420 represent the quantities of colorless intermediates generated during the initial phase of the MR and the quantities of browning products formed in the later phase, respectively.
